# Practice and attitude of general practitioners towards initiating isotretinoin for acne vulgaris in Fars province, Iran: cross-sectional study

**DOI:** 10.1186/s12875-023-02260-w

**Published:** 2024-01-13

**Authors:** Parisa Hosseinpour, Ghazal Gholamabbas, Fatemehsadat Pezeshkian, Amirhossein Erfani, Reza Shahriarirad, Ahmad Reza Parhizkar

**Affiliations:** 1 School of Medicine, Islamic Azad University, Kazeroun branch, Kazeroun, Iran; 2 Student research committee, Shiraz University of Medical Sciences, Shiraz, Iran; 3 Thoracic and Vascular Surgery Research Center, Shiraz University of Medical Sciences, Shiraz, Iran

**Keywords:** Attitude, General practitioners, Isotretinoin, Practice, Primary care

## Abstract

**Background:**

Since general practitioners manage acne-related referrals, there needs to be more information in Iran about how drugs such as Isotretinoin are prescribed and the treatment plan. Thus, this study aimed to evaluate general practitioners s’ practices and attitudes in prescribing Isotretinoin for acne vulgaris in primary care.

**Methods:**

This web-based cross-sectional descriptive study was conducted using two questionnaires designed with the target population of GPs working in Fars province in 2021 regarding the prescription of Isotretinoin. Moreover, demographic information, questions about interest in dermatology, and participation in dermatology workshops were gathered.

**Results:**

A total of 308 complete questionnaires were obtained. According to our results, 85 (27.6%) GPs prescribed Isotretinoin in primary care. Based on our results, higher age (OR: 1.042; CI95%: 1.013–1.072; *P*-value:0.004) and attending dermatological courses (OR: 3.280; CI95%: 1.592–6.755; *P*-value:0.001) were significantly correlated with more frequent Isotretinoin administration. Among GPs who do not prescribe Isotretinoin, the most common causes are concerns about liver dysfunction (54.7%), teratogenic concerns (37.2%), and lack of familiarity with the drug (31.4%) respectively.

**Conclusion:**

The results of this study depicted the reluctance of most physicians to prescribe Isotretinoin and factors such as taking part in supplementary courses under the supervision of dermatologists and following national guidelines that could encourage them to prescribe Isotretinoin.

## Introduction

Acne vulgaris is a prevalent and persistent inflammation of the pilosebaceous unit that often arises during puberty and may or may not subside as puberty comes to a close [1]. While males are more prone to severe forms during puberty, females tend to develop acne vulgaris more frequently as they age [2]. Acne vulgaris is most commonly found in areas with abundant sebaceous glands, such as the face, proximal upper extremity, neck, and trunk [3]. The severity of the disease can range from self-limiting types that resolve without scarring to severe forms that leave complications such as atrophic scars or post-inflammatory hyperpigmentation. The complications can negatively impact self-image and quality of life, leading to higher rates of depression, anxiety, and even suicidal thoughts [4].

The primary objective of treatment is to enhance appearance while minimizing the potential for scarring and psychological distress [3]. Topical agents are the preferred approach for addressing mild to moderate acne due to their lower incidence of adverse reactions. Systemic agents may be necessary in cases where topical treatments are ineffective or when severe acne is present [2].

Isotretinoin is a prescription-only drug derived from vitamin A. It is effective in treating various causes of acne by reducing inflammation and comedones and decreasing follicular keratinization, sebum production, neutrophil chemotaxis, and the amount of *Cutibacterium acnes* in the follicles [5]. Isotretinoin is primarily prescribed for severe cases of acne vulgaris, but it may also be recommended for moderate forms where there is evidence of scarring, previous treatment failure, or psychological distress related to acne [3, 6, 7]. Adverse effects of this medication are typically short-lived and vary depending on the dosage, including pain, mucocutaneous dryness, and erythema [8]. Furthermore, it’s important to note that severe side effects such as teratogenicity, elevated cholesterol levels, depressive symptoms, and liver dysfunction have been associated with its use [9]. Ultimately, the benefits of Isotretinoin in treating acne should be carefully considered alongside the potential risks [10, 11].

Although Isotretinoin has the potential to treat acne effectively, there are various controversial opinions surrounding this medication that are often based on insufficient data or anecdotes [9]. In light of this, our study aimed to examine the knowledge and attitudes of general practitioners (GPs) in Fars province, Iran, regarding the prescription of Isotretinoin for acne vulgaris. This investigation is vital for enhancing patient health and preventing undesirable side effects.

## Methods and materials

### Study design and participants

This study was a cross-sectional online survey among GPs practicing in Fars province, Iran, conducted in August the first 2021 for three weeks to investigate their knowledge and attitude regarding the administration of Isotretinoin for acne vulgaris. Inclusion criteria included GPs currently working in Fars province who consented to participate in the study, and those who did not have consent were excluded.

The minimum required sample size for our study was calculated based on a survey by Carmody et al. by assuming a confidence interval of 95% and a margin of error of 0.5% to be 211, taking into consideration that 7680 GPs are working in Fars province (1.6 GPs per 1000 people) [12].

### Study area

Fars province is one of the thirty-one provinces of Iran located in the southwest of the country with a population of over 4.7 million; it is considered to be the medical center of the south of Iran with many referrals from neighboring provinces and countries around the Persian Gulf such for various medical and health matter. As one of the pioneers in implementing the referral-based healthcare program in Iran, Fars province has an extensive network of primary healthcare facilities and secondary and tertiary referral hospitals across the region providing health services to its residents.

### Questionnaire and data collection

By reviewing related documents and articles, this study implemented two questionnaires based on the survey conducted by Carmody et al., one for GPs who prescribed Isotretinoin and another for those who did not [12]. Demographic information such as age and gender, along with related questions regarding interest in the field of dermatology, being a member of a dermatology association, participation in dermatology courses, and work experience under the supervision of a dermatologist, were included at the beginning of each questionnaire. Moreover, fifteen questions in those who prescribe Isotretinoin in their practice about indication and its management (Table 1) were inquired and in those who do not prescribe Isotretinoin, ten questions regarding their hesitancy and concerns as well as conditions in which they would support its prescription were also included (Table 2).

**Table 1:** Responses of general practitioners who prescribe Isotretinoin toward their management and practice

Question	Answer	Frequency (%); *n = 85*
In the past year (2020), how many patients did you prescribe Isotretinoin for?	0–10	77 (90.6)
	10–50	6 (7.1)
	50–100	2 (2.4)
	More than 100	0
What are your main reasons for starting Isotretinoin in a primary care setting?	Interest in treating patients with dermatologic disorders	51 (60)
	The long wait for dermatologist visits	13 (15.3)
	Experience in working in an aesthetic medicine clinic	8 (9.4)
	No response to prior treatments	5 (6.0)
	Financial incentive	3 (3.5)
	Indication for resistant nodulocystic acne	1 (1.2)
	According to my previous experiences, I prescribe for those with severe acne	1 (1.2)
	None of the above	16 (18.8)
What did you prescribe Isotretinoin for?	Moderate to severe acne	53 (62.4)
	Nodulocystic acne	46 (54.1)
	No response to oral antibiotics	41 (48.2)
	Patient’s request	19 (22.4)
	Acne accompanied by an ulcer	5 (5.9)
	Psychologic complications due to having acne	1 (1.2)
Which gender group would you prescribe Isotretinoin for?	Females	22 (25.9)
	Males	7 (8.2)
	Both	56 (65.9)
What are the reasons you refuse to prescribe Isotretinoin?	Labs indicating liver failure	79 (92.9)
	Untrustworthy contraceptive methods	61 (71.8)
	Alcoholism	45 (52.9)
	Central nervous system disorders	29 (34.1)
	Current depression	23 (27.1)
	Previous history of depression	12 (14.1)
	Abnormal lipid profile	4 (4.8)
	Pregnancy and fertile groups	1 (1.2)
How would you decide on Isotretinoin dosage?	Based on weight	40 (47.1)
	General starting dose	39 (45.9)
	Based on acne severity	34 (40.0)
	Based on gender	4 (4.7)
At what intervals would you visit female patients?	Every month	52 (61.2)
	Every two months	12 (14.1)
	Every three months	12 (14.1)
	Only the first session	4 (4.7)
	None of the above	4 (4.7)
	With intervals of more than three months	1 (1.2)
At what intervals would you visit male patients?	Every month	41 (48.2)
	Every two months	15 (17.6)
	Every three months	13 (15.3)
	Only the first session	4 (4.7)
	With intervals of more than three months	4 (4.7)
	None of the above	4 (4.7)
In what intervals would you request pregnancy tests?	Each month before prescription	44 (51.8)
	Only the first session	21 (24.7)
	With intervals of more than a month	12 (14.1)
	Never	6 (7.1)
	In the case of pregnancy, I would not prescribe	1 (1.2)
	I have not had a female patient before	1 (1.2)
What methods would you choose for contraception?	Barrier contraception	43 (50.6)
	Oral contraceptive drugs (OCP)	24 (28.2)
	Both intra-uterine devices (IUD) and OCP	21 (24.7)
	Long-term contraceptive methods like progesterone injections or IUD	14 (16.5)
	I would not prescribe anything	12 (14.1)
	None of the above	6 (7.1)
Would you refer patients with a previous history of depression to a specialist before starting Isotretinoin?	No	33 (38.8)
	Yes	22 (25.9)
	If there is a possibility of having depression, I would not prescribe Isotretinoin	20 (23.5)
	It depends on the patient	10 (11.8)
Would you request blood tests before starting Isotretinoin?	Yes	79 (92.9)
	No	6 (7.1)
What type of blood tests would you request?	Liver function tests	79 (92.9)
	Lipid profile	57 (67.1)
	Complete blood count	57 (67.1)
	Fasting blood sugar	36 (42.4)
	Thyroid function tests	28 (32.9)
After starting treatment, at what interval would you request blood tests?	Every month	31 (36.5)
	Once after 6–8 weeks	7 (8.2)
	Every two months	14 (16.5)
	Every three months	16 (18.8)
	Intervals of more than three months	8 (9.4)
	None of the above	1 (1.2)
	One month after, then every two months	1 (1.2)
For how long would you prescribe Isotretinoin in each visit?	Less than a month	11 (12.9)
	One to three months	30 (35.3)
	Three to six months	35 (41.2)
	Six to nine months	3 (3.5)
	Nine to twelve months	4 (4.7)
	More than twelve months	2 (2.4)

**Table 2:** Responses of general practitioners regarding not prescribing Isotretinoin in their practice

Question	Response	Frequency (%); N = 223
Why would you not prescribe Isotretinoin?	Not being aware that GPs have permission to prescribe it	58 (74)
	Worries regarding liver dysfunction	122 (54.7)
	Worries regarding its teratogenicity	83 (37.2)
	Lack of knowledge of the drug	70 (31.4)
	Worries regarding abnormal lipid profile	46 (20.6)
	I am not interested	47 (21.1)
	Being wary of the legal complaints	37 (16.6)
	No coverage by insurance companies	23 (10.3)
	Worries regarding mucocutaneous dryness	22 (9.9)
	Worries regarding workload	10 (4.5)
	Being wary of suicidality	8 (3.6)
	Not having relevant patients	8 (3.2)
	I believe only dermatologists should prescribe it	10 (3.1)
	There was no need for my patients to start Isotretinoin	7(2.8)
	Not having accurate follow-up	2 (0.8)
	Drug side effects	2 (0.8)
	Not in my experience field	1 (0.4)
Are you interested in prescribing Isotretinoin in your future primary care visits?	No	114 (51.1)
	Yes	109 (48.9)
What would persuade you to prescribe Isotretinoin?	Guideline from health care systems	111 (49.8)
	Complementary educations	103 (46.2)
	The guidance of a dermatologist, if needed	103 (46.2)
	Proper support from the Food and Drug Administration (FDA) and forensic medicine	67 (30)
	Financial aid	16 (7.2)
	Proper follow-up	2 (0.8)
	Proper knowledge of the drug	1 (0.4)
	Higher efficacy compared to other drugs of choice	1 (0.4)
	Patient’s request	1 (0.4)
	Indication for starting Isotretinoin	1 (0.4)
	None of the above	30 (13.4)
Do you believe that Isotretinoin should only be administered by a specialist?	Yes	122 (54.7)
	No	101 (45.3)
In your opinion, what are the requirements for GPs who prescribe Isotretinoin?	Complementary education in Dermatology	105 (47.1)
	Previous experience in working under a dermatologist supervision	82 (36.8)
	being a member of dermatology associations	21 (9.4)
	Interest in the dermatology field	18 (8.1)
	Being aware of the drug information (indication, blood tests, and follow-up)	7 (2.8)
	Obeying the guideline	1 (0.4)
	Insufficient education in this regard	1 (0.4)
	No response in first-line treatment options	1 (0.4)
	None of the above	76 (34)
Are you aware that Isotretinoin is being prescribed in primary care centers?	Yes	109 (48.9)
	No	114 (51.1)
Do you believe that primary care settings can prescribe Isotretinoin safely?	Yes	105 (47.1)
	No	118 (52.9)
Do you believe there are safety measures in the primary care setting for prescribing Isotretinoin?	Yes	134 (60.1)
	No	89 (39.9)
Would it be beneficial if Isotretinoin were used vastly in primary care settings?	Yes	107 (48)
	No	116 (52)
Would the specialists support Isotretinoin usage in primary care settings?	Yes	55 (24.7)
	No	168 (75.3)

The questionnaires were designed online via Porsline *(*https://www.porsline.ir/*)* with fixed-choice responses, and the questionnaire link was distributed on social networks (e.g., Email, Telegram, WhatsApp, and Instagram) related to GPs working in Fars province.

### Statistical analysis

The obtained information was statistically analyzed using SPSS version 26 (IBM, USA). The normal distribution of data was checked with the Komarov-Shapiro test. Analysis was performed by calculating the data’s mean, percentage (%), and ± standard deviation (SD). The Chi-square and T-test were used to compare the variables, and the significance level of the test was considered to be 0.05. We also utilized multiple regression analysis to evaluate factors correlated with Isotretinoin administration based on the participants’ variables.

## Results

Among 668 GPs who viewed the questionnaire, we received 308 completed forms (46%). The mean age of the participants was 31.8 ± 8.8 (range: 24–67), and 145 (47.1%) were male. Among them, 153 (49.7%) were interested in the field of dermatology. Also, 10 participants (2.3%) were members of dermatology associations, and 47 (15.3%) had completed dermatology training courses. In addition, 31 participants (10.1%) had work experience under the supervision of a dermatologist. Based on the results of our study, 85 (27.6%) of GPs prescribed isotretinoin in primary care. Table 3 demonstrates the overall features of the participants in our study and compares the characteristics of GPs who prescribe and those who do not prescribe Isotretinoin.

**Table 3:** Overall demographic features, interests, and experience of general practitioners in the dermatology field in our study based on the prescription of Isotretinoin in their practice

Variables		Total; *N = 308*	Isotretinoin prescription		*P*-value*
			**No;** *n = 223*	**Yes**; *n = 85*	
Age (year); mean ± standard deviation		31.81 ± 8.81	30.75 ± 8.02	34.56 ± 10.14	**0.002**
Gender; n (%)	*Male*	145 (47.1)	107 (73.8)	38 )26.2)	0.607
	*Female*	163 (52.9)	116 (71.2)	47 (28.8)	
Interest in the field of dermatology; n (%)	*No*	153 (49.7)	119 (77.8)	34 (22.2)	**0.036**
	*Yes*	155 (50.3)	104 (67.1)	51 (32.9)	
Being a member of dermatology associations; n (%)	*No*	298 (96.8)	218 (73.2)	80 (26.8)	0.146
	*Yes*	10 (3.2)	5 (50)	5 (50)	
Participation in dermatology courses; n (%)	*No*	261 (84.7)	202 (77.4)	59 (22.6)	**< 0.001**
	*Yes*	47 (15.3)	21 (44.7)	26 (55.3)	
Work experience under dermatologist supervision; n (%)	*No*	277 (89.9)	208 (75.1)	69 (24.9)	**0.002**
	*Yes*	31 (10.1)	15 (48.4)	16 (51.6)	

Bold values indicate a significant association

*Independent sample t-test or Chi-Square/Fisher’s exact test

It is worth mentioning that older physicians tend to prescribe Isotretinoin more than younger ones (*P* value = 0.002). In addition, those who were interested in the field of dermatology (OR: 1.71; CI95%: 1.03–2.85; *P* value = 0.036) and also those who participated in dermatology courses (OR: 4.23; CI95%: 2.22–8.07; *P* value < 0.001) and had work experience under the supervision of a dermatology specialist (OR: 3.22; CI95%: 1.51–6.84; *P* value = 0.002) were more willing to prescribe Isotretinoin.

We further investigated higher Isotretinoin administration based on multiple regression analysis among the participants’ factors including age, sex, dermatology field interest, participation in dermatology courses, work experience under the supervision of a dermatologist, and being a member of a dermatology association. Based on our results, only higher age (OR: 1.042; CI95%: 1.013–1.072; *P*-value:0.004) and attending dermatological courses (OR: 3.280; CI95%: 1.592–6.755; *P*-value:0.001) were significantly correlated with more frequent Isotretinoin administration.

The responses regarding the questionnaire on isotretinoin prescription are demonstrated in Table 1.

According to the answers, most GPs (90.6%) had prescribed Isotretinoin to less than ten people. Also, the interest in treating patients with skin problems (60%) is one of the main reasons for prescribing this medication. The most indications for prescribing are moderate to severe acne (62.4%) and nodulocystic acne (54.1%). Moreover, 22.4% of the participants stated the reason for the prescription was the patient’s request, and according to the analyzed data, Isotretinoin was mainly prescribed for women.

**Fig. 1:**
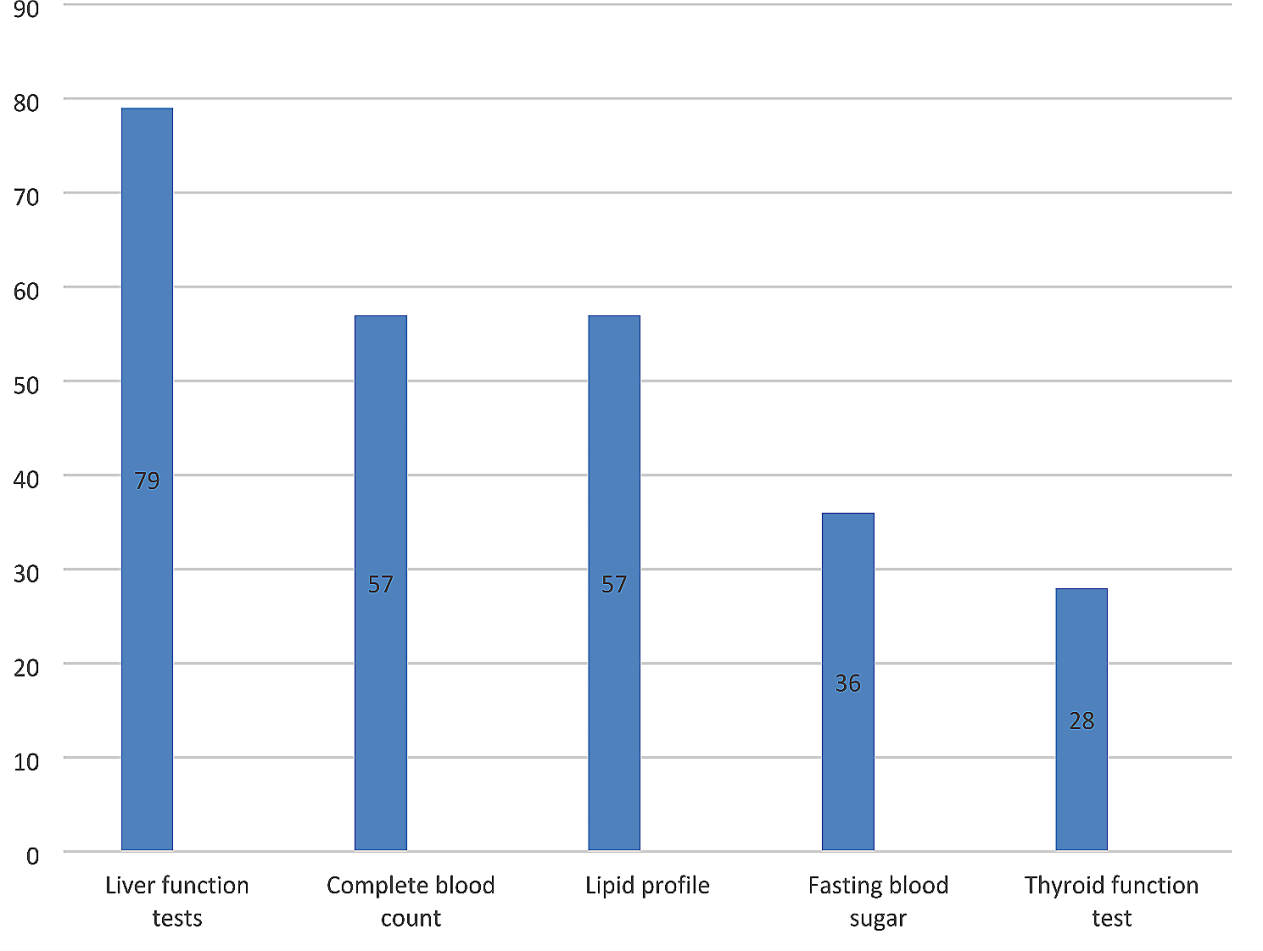
Laboratory results evaluated by general practitioners regarding blood examination prior to isotretinoin prescription

Disturbance in liver function tests (92.9%) and untrustworthy contraceptive methods (71.8%) were the major factors that prevented isotretinoin prescription. In most cases, pregnancy tests were performed once a month before each prescription. Also, barrier contraceptives were the most prescribed contraceptive method. 38.8% of participants were observed not to refer the patient to a psychiatrist if there was a history of depression before starting treatment, and only 14.1% refused to prescribe Isotretinoin if there was a history of depression. Almost all physicians evaluated liver function tests among blood tests. It is worth mentioning that evaluating tests such as complete blood count (CBC), fasting blood sugar (FBS), and thyroid function test (TFT) also had a high proportion. The distribution of blood tests is demonstrated in Fig. 1.

Among the participants, 223 (72.4%) did not prescribe Isotretinoin, demonstrating their responses in this regard in Table 2. Concerns regarding liver dysfunction (54.7%), teratogenicity (37.2%), and drug unfamiliarity (31.4%) were, respectively, the most common reasons among physicians not prescribing Isotretinoin.

Furthermore, due to the reluctance of the majority of the physicians to prescribe this drug, the recommendations and instructions of the relevant organizations (49.8%), training in this field (46.2%), and the guidance and support of dermatologists if needed (46.2%) were the essential encouraging factors for prescribing this drug. Also, these physicians believed that those who completed postgraduate education courses in dermatology (47.1%) or had work experience under the supervision of dermatologists (36.8%) would be more eligible to prescribe the drug than others. Additionally, physicians who did not prescribe Isotretinoin believed that their dermatologist colleagues did not support the widespread use of Isotretinoin in the primary care units.

## Discussion

The overall results indicated that most of the GPs do not resort to prescribing Isotretinoin. Notably, interest in the dermatology field, dermatologic courses partake, experience working under a dermatologist’s supervision, and older age, all contributed significantly to prescribing Isotretinoin more in primary care units. Furthermore, it was observed that those who did prescribe Isotretinoin tended to do it rather scarcely (less than ten times each year). Notably, while most GPs fulfilled in prescribing Isotretinoin based on indications, a number of them also prescribed it upon the patient’s request. Almost all participants were aware of requesting necessary lab data for patients, such as LFT or lipid profile; however, many also requested unnecessary lab work, such as TFT, CBC, or FBS. Lastly, several GPs overlooked enlightening patients regarding using reliable methods of contraception.

Most GPs who refrained from prescribing Isotretinoin were apprehensive due to the adverse effects, such as teratogenicity or impaired liver function, and the lack of a well-ordered system to monitor them. Most of them (51.1%) demonstrated an unwillingness to prescribe Isotretinoin. They believed that it is unsafe to prescribe Isotretinoin in primary health units. Neither the patients nor the physicians would profit from it. At the same time, dermatologists would not support its prescription in primary health care, and prescribing should be based upon dermatologic guidelines and under their supervision. In Iran, the need for a nationally organized guideline related to the prescription of Isotretinoin has made general physicians uncertain about the prescription of this drug as to why the majority believed that this drug should only be prescribed by a specialist. Furthermore, they believed that the broad prescription of these drugs was not in the patient’s interest. Meanwhile, the existence of national and pharmaceutical guidelines in Ireland encourages Irish GPs to be more willing to prescribe this drug with support from specialists and believe this drug can be prescribed without risks in patients in primary care [12]. While the debate continues regarding whether patients with acne vulgaris should primarily be managed by GPs in an outpatient setting or by dermatology specialists, the appropriate approach largely depends on the healthcare system’s workload and accessibility within each respective country. One feasible method suggests that patients undergo their initial consultation with a specialist for a comprehensive examination and the initiation of treatment, particularly considering the potential link between acne vulgaris and various dermatoendocrinological disorders [13]. This approach aims to reduce misdiagnoses and overlooked cases. Given the diverse nature of acne, potentially linked to syndromes (PAPASH, SAPHO) or dermatoendocrinological disorders (PCOS, HAIR-AN), the decision to start isotretinoin emphasizes the need for a dermatologist’s examination. This is crucial to avoid missing syndromic components or exacerbating underlying conditions. Such a strategy is practical in settings where an unreliable system for monitoring adverse effects and laboratory work impedes GPs from prescribing isotretinoin. Nonetheless, its success hinges on the implementation of a well-organized system for GP follow-up and patient tracking, thereby ensuring a secure and effective treatment process. In high-volume settings, it becomes imperative for GPs to deepen their understanding of isotretinoin, encompassing its potential side effects and associated conditions. By raising awareness of concurrent diseases and potential side effects, GPs can facilitate prompt and early referrals to specialists, thereby creating a safe and structured environment for acne treatment. Importantly, this approach also serves to alleviate the workload of specialists in Iran.

Since acne is one of the chief complaints that patients present with in primary health units, and considering the GPs’ role in treating acne in primary care settings, it is crucial that they are provided with sufficient access and information regarding Isotretinoin. The prescription of Isotretinoin worldwide varies depending on their health care system’s policy. In the UK, with a referral-based health system, this drug can only be prescribed by a specialist, whereas in other countries, such as the Netherlands, GPs can prescribe this drug according to the guidelines. Moreover, in New Zealand, primary care physicians have been prescribing Isotretinoin without funding restrictions since 2009, facilitating access to this medication for low-income groups that geographically had less access to specialists. GPs participated in 58% of isotretinoin prescriptions in 2012 in New Zealand [14–17]. In Iran, Isotretinoin is not currently covered by insurance. However, only 10% of participants who did not prescribe this drug stated that the lack of insurance prevents the prescription. This may be because the price of this drug in Iran is lower than the average in other countries. Conclusively, insurance policies should be reevaluated, and further insurance coverage should be planned for the doctors who took dermatologic courses and know the drug instructions.

Physicians who prescribed Isotretinoin were observed to request unnecessary lab tests for the patients, including thyroid function tests, CBC, and FBS, in addition to necessary ones such as liver function tests and lipid profiles, and most physicians performed these tests once a month. Accordingly, Carmody et al. observed that most patients on Isotretinoin underwent more blood tests than was recommended, while some patients did not have enough tests done [12]. An evidence-based approach to Isotretinoin laboratory monitoring indicates that baseline lipid panel and LFT should be taken, and the approach to monitoring intervals defers from that. If the baseline laboratory data are normal, it is recommended that the tests be repeated two months after therapy, and if that is in the normal range, no further testing is required. However, if the results fail to be normal, continuing monitoring with dosage alteration is needed [18]. All in all, failure to comply with relevant guidelines by physicians in conducting lab tests costs both the patients and the healthcare system.

Considering that most of the doctors who prescribed Isotretinoin were aware of the side effects of this drug on liver function and blood lipid profile, many of them were not aware of the possibility of its association with mental problems. This result was in line with a previous study on Irish doctors who neglected to consider psychiatry issues when prescribing Isotretinoin [12]. While the association between Isotretinoin and teratogenicity is clinically evident, its connection to mental complications is a matter of debate. In the 1990s, with the extent of using Isotretinoin, neuropsychiatric side effects emerged in the shapes of anxiety and depression. Suicidal thoughts, impulsivity, and the Food and Drug Administration (FDA) implemented a warning regarding this new adverse reaction [19]. There is a controversial relationship between isotretinoin therapy for acne and depression. Mental alteration has been reported in patients who used Vitamin A, Etretinate, or Isotretinoin, while no reports have been made regarding other retinoids such as Bexarotene [20, 21]. A survey of 1419 people concluded that 17.2% of people treated with this drug required mental health counseling services [22]. Meanwhile, several studies did not demonstrate a correlation between Isotretinoin and an increased risk of depression and, in addition, revealed that treating acne improved symptoms of depression [23]. However, a global study into Isotretinoin revealed that Isotretinoin improved depression while it deteriorated suicidal ideations [24]. Moreover, a recent meta-analysis by Tan et al. including more than 1,600,000 participants revealed a low absolute risk and no increase relative risk of psychiatric disorders and suicide attempts in people taking isotretinoin. In reality, those who took isotretinoin had a lower risk of suicide attempts in 2 to 4 years following administration [25]. Nevertheless, while recent population base studies had promising results regarding the effect of Isotretinoin on psychiatric disorders, clinicians should be vigilant and monitor patients in a holistic approach for signs of mental illnesses.

Among the limitations of this study, the following points can be mentioned. The number of GPs surveyed in this research is undersized and may only represent some GPs working in the province. For this reason, studies with larger populations are recommended in the future. In this study, the mean age of GPs were young, probably because the questionnaire was online, which may not include the work experience of more experienced and older doctors. Physicians who currently prescribe Isotretinoin or have a particular interest in dermatology were more likely to respond so that it could cause some bias.

## Conclusion

According to the findings of this study, many physicians are hesitant to prescribe this particular medication. Potential solutions to address this issue include increasing physician awareness through additional training under dermatology specialists’ guidance and establishing national guidelines to guide their prescribing decisions. Additionally, healthcare organizations in Iran should develop more comprehensive monitoring protocols to ensure that lab tests and patient follow-ups are conducted in accordance with established guidelines, thereby instilling greater confidence among general practitioners when it comes to prescribing Isotretinoin.

## Data Availability

The present study’s findings are available on request from the corresponding author. They are not publicly available due to privacy and ethical restrictions.

## Abbreviations

CBC: Complete Blood Count
FBS: Fasting Blood Sugar
FDA: Food and Drug Administration
GP: General Practitioner
IUD: Intra Uterine Device
LFT: Liver Function Tests
OCP: Oral Contraceptive
TFT: Thyroid Function Tests

## References

[1] Bhate K, Williams HC (2013). Epidemiology of acne vulgaris. Br J Dermatol.

[2] Lynn DD, Umari T, Dunnick CA, Dellavalle RP (2016). The epidemiology of acne vulgaris in late adolescence. Adolesc Health Med Ther.

[3] Leung AK, Barankin B, Lam JM, Leong KF, Hon KL. Dermatology: how to manage acne vulgaris. Drugs Context. 2021;10. 10.7573/dic.2021-8-6 PMC8510514 34691199

[4] Eichenfield DZ, Sprague J, Eichenfield LF (2021). Management of Acne Vulgaris: a review. JAMA.

[5] Khiali S, Gharekhani A, Entezari-Maleki T, Isotretinoin (2018). A review on the utilization pattern in pregnancy. Adv Pharm Bull.

[6] Sadeghzadeh-Bazargan A, Ghassemi M, Goodarzi A, Roohaninasab M, Najar Nobari N, Behrangi E (2021). Systematic review of low-dose isotretinoin for treatment of acne vulgaris: focus on indication, dosage, regimen, efficacy, safety, satisfaction, and follow up, based on clinical studies. Dermatol Ther.

[7] Kraft J, Freiman A (2011). Management of acne. CMAJ.

[8] Han JJ, Faletsky A, Barbieri JS, Mostaghimi A (2021). New Acne therapies and updates on Use of Spironolactone and Isotretinoin: a narrative review. Dermatol Ther (Heidelb).

[9] Landis MN (2020). Optimizing Isotretinoin Treatment of Acne: update on current recommendations for monitoring, Dosing, Safety, adverse effects, Compliance, and outcomes. Am J Clin Dermatol.

[10] Zane LT, Leyden WA, Marqueling AL, Manos MM (2006). A population-based analysis of laboratory abnormalities during isotretinoin therapy for acne vulgaris. Arch Dermatol.

[11] Wysowski DK, Pitts M, Beitz J (2001). An analysis of reports of depression and Suicide in patients treated with isotretinoin. J Am Acad Dermatol.

[12] Carmody K, Rouse M, Nolan D, Quinlan D (2020). GPs’ practice and attitudes to initiating isotretinoin for acne vulgaris in Ireland: a cross-sectional questionnaire survey in primary care. Br J Gen Pract.

[13] Chen W, Obermayer-Pietsch B, Hong JB, Melnik BC, Yamasaki O, Dessinioti C (2011). Acne-associated syndromes: models for better understanding of acne pathogenesis. J Eur Acad Dermatol Venereol.

[14] Goodfield MJD, Cox NH, Bowser A, McMillan JC, Millard LG, Simpson NB (2010). Advice on the safe introduction and continued use of isotretinoin in acne in the U.K. 2010. Br J Dermatol.

[15] Moodie P, Jaine R, Arnold J, Bignall M, Metcalfe S, Arroll B (2011). Usage and equity of access to isotretinoin in New Zealand by deprivation and ethnicity. N Z Med J.

[16] Bruinsma MDRW, Jaspar AHJ, Van der Zee HH, Van Vugt SF, Verhoeven ICL, Verstappen V en, Wiersma TJ. NHG: The Dutch College of General Practitioners. NHG Standard. Acne. 2017 [Available from: https://richtlijnen.nhg.org/standaarden/acne.

[17] Oakley A (2013). Managing acne in primary care. BPJ.

[18] Hansen TJ, Lucking S, Miller JJ, Kirby JS, Thiboutot DM, Zaenglein AL (2016). Standardized laboratory monitoring with use of isotretinoin in acne. J Am Acad Dermatol.

[19] Bremner JD (2021). Isotretinoin and neuropsychiatric side effects: continued vigilance is needed. J Affect Disord Rep.

[20] Zaenglein AL (2018). Acne Vulgaris. N Engl J Med.

[21] Oliveira JM, Sobreira G, Velosa J, Telles Correia D, Filipe P (2018). Association of Isotretinoin with Depression and Suicide: a review of current literature. J Cutan Med Surg.

[22] Friedman T, Wohl Y, Knobler HY, Lubin G, Brenner S, Levi Y (2006). Increased use of mental health services related to isotretinoin treatment: a 5-year analysis. Eur Neuropsychopharmacol.

[23] Huang YC, Cheng YC (2017). Isotretinoin treatment for acne and risk of depression: a systematic review and meta-analysis. J Am Acad Dermatol.

[24] Kridin K, Ludwig RJ (2023). Isotretinoin and the risk of psychiatric disturbances: a global study shedding new light on a debatable story. J Am Acad Dermatol.

[25] Tan NKW, Tang A, MacAlevey NCYL, Tan BKJ, Oon HH. Risk of Suicide and psychiatric disorders among isotretinoin users: a meta-analysis. JAMA Dermatol 2023;29:e234579. 10.1001/jamadermatol.2023.4579 PMC10687715 38019562

